# Changes in abscisic acid metabolism in relation to the maturation of grapevine (*Vitis vinifera* L., cv. Mencía) somatic embryos

**DOI:** 10.1186/s12870-020-02701-z

**Published:** 2020-10-23

**Authors:** Yosvanis Acanda, Óscar Martínez, María Jesús Prado, María Victoria González, Manuel Rey

**Affiliations:** 1 Departamento de Biología Vegetal y Ciencia del Suelo, Universidad de Vigo, Campus Universitario, 36310 Vigo, Spain; 2 Present Address; Department of Plant Pathology, Citrus Research and Education Center, UF-IFAS, 700 Experiment Station Rd, Lake Alfred, FL 33850 USA; 3 Departamento de Biología Funcional, Universidad de Santiago de Compostela, Campus Sur, 15872 Santiago de Compostela, Spain; 4 CITACA, Agri-Food Research and Transfer Cluster, Campus da Auga, Universidad de Vigo, 32004 Ourense, Spain

**Keywords:** Somatic embryogenesis, qPCR, Reference genes, Cellulose semipermeable membrane, ABA metabolism, Gene expression

## Abstract

**Background:**

Somatic embryogenesis in grapevines is a complex process that depends on many physiological and genetic factors. One of its main limitations is the process of precocious germination of the somatic embryos in differentiation medium. This process lowers plant conversion rates from the somatic embryos, and it is probably caused by a low endogenous abscisic acid (ABA) content.

**Results:**

Precocious germination of the somatic embryos was successfully avoided by culturing grapevine cv. Mencía embryogenic aggregates over a semipermeable membrane extended on top of the differentiation medium. The weekly analysis of the endogenous ABA and ABA-glucosyl ester (ABA-GE) contents in the aggregates showed their rapid accumulation. The expression profiles of 9-*cis*-epoxycarotenoid dioxygenase (*VvNCED1*), 8′-hydroxylase (*VvHyd2*), UDP-glucosyltransferase (*VvUGT*) and β-glucosidase (*VvBG2*) genes in grapevine revealed that the occurrence of a first accumulation peak of endogenous ABA in the second week of culture over the semipermeable membrane was mainly dependent on the expression of the *VvNCED1* gene. A second increase in the endogenous ABA content was observed in the fourth week of culture. At this point in the culture, our results suggest that of those genes involved in ABA accumulation, one (*VvNCED1*) was repressed, while another (*VvBG2*) was activated. Similarly, of those genes related to a reduction in ABA levels, one (*VvUGT*) was repressed while another (*VvHyd2*) was activated. The relative expression level of the *VvNCED1* gene in embryogenic aggregates cultured under the same conditions and treated with exogenous ABA revealed the significant downregulation of this gene.

**Conclusions:**

Our results demonstrated the involvement of ABA metabolism in the control of the maturation of grapevine somatic embryos cultured over a semipermeable membrane and two important control points for their endogenous ABA levels. Thus, subtle differences in the expression of the antagonistic genes that control ABA synthesis and degradation could be responsible for the final level of ABA during the maturation of grapevine somatic embryos in vitro. In addition, the treatment of somatic embryos with exogenous ABA suggested the feedback-based control of the expression of the *VvNCED1* gene by ABA during the maturation of grapevine somatic embryos.

**Supplementary information:**

**Supplementary information** accompanies this paper at 10.1186/s12870-020-02701-z.

## Background

Early embryogenesis in angiosperms lasts from the unicellular stage to the heart stage and comprises a period of extensive cell division controlled by high levels of indole-3-acetic acid (IAA) and low levels of abscisic acid (ABA). Mid-embryogenesis includes the heart stage to the torpedo stage, ending with rapidly increasing ABA levels. It is likely that this increase in ABA content induces important metabolic changes that allow the storage of reserves as well as embryo desiccation and dormancy during late embryogenesis [1]. It has been shown that the application of exogenous ABA enhanced the tolerance of alfalfa somatic embryos to desiccation, thus improving the quality of the plants converted from these embryos [2, 3]. In Norway spruce, Vondrakova et al. (2018) [4] observed a peak accumulation of ABA in maturing somatic embryos and another peak of ABA-glucosyl ester (ABA-GE) at desiccation. During somatic embryogenesis in cotton [5], the transcriptional activation of stress responses was observed, with enhanced expression levels of stress-related genes, including several that are involved in ABA biosynthesis and signaling. Additionally, the maturation of tulip somatic embryos requires high ABA levels [6].

In our lab, we established an efficient protocol for somatic embryogenesis from stamen filaments in grapevine (cv. Mencía) using thidiazuron (1-phenyl-3-(1,2,3-thidiazol-5-yl)urea) and 2,4-dichlorophenoxyacetic acid (2,4-D) [7]. When using this protocol, we found that the somatic embryos developed asynchronously, and a significant proportion of them germinated precociously when globular somatic embryo aggregates were cultured as small-sized inocula (0.1–0.5 mg fresh weight) in DM1 differentiation medium [7] for 4 weeks. We demonstrated that precocious germination of somatic embryos during their culture in differentiation medium negatively affected their further conversion to plantlets in several grapevine cultivars [8, 9], so we expended effort to try to prevent this phenomenon. By culturing larger inocula (50–80 mg fresh weight), precocious germination was avoided in Mencía somatic embryos [7], although a significant proportion of embryos remained at an early developmental stage (torpedo shape), suggesting that cultures need over 4 weeks on differentiation medium to reach the cotyledonary stage. This result may have been due to the slower diffusion of nutrients and growth regulators and slower water uptake in the embryos cultured as large inocula [7]. In grapevine, the proper maturation of somatic embryos has been related with their accumulation of reserve products [10, 11] and desiccation [1]. In this context, one approach to obtaining mature somatic embryos could be the reduction of water availability by, for example, culturing the somatic embryos on top of a cellulose acetate semipermeable membrane. This procedure has been previously used to control the maturation of somatic embryos in citrus and olive [12, 13]. In avocado (*Persea americana*), the culture of embryogenic calli on top of cellulose acetate membranes increased the number of well-developed mature somatic embryos and improved their further germination [14].

It has been demonstrated that the exogenous application of ABA can improve the maturation of grapevine somatic embryos [15] because ABA slows the growth of somatic embryos and improves their conversion to plants by preventing precocious germination. It has been shown that the absence of an accumulation peak of endogenous ABA may be responsible for the abnormal maturation and precocious germination of grapevine somatic embryos [9, 16]. In higher plants, ABA is synthesized from an oxidative cleavage of the epoxy-carotenoids 9-*cis* neoxanthin and 9-*cis* violaxantin to produce xanthoxin, which is subsequently converted to ABA in the cytosol. This reaction is catalyzed by the 9-*cis*-epoxycarotenoid dioxygenase (NCED) family of enzymes that was first described as viviparous14 (VP14) in maize, and the reaction is considered the first and most important regulatory step in the ABA biosynthetic pathway [17]. However, the concentration of ABA and hence its regulatory role is a consequence of the rate of ABA biosynthesis and catabolism [18]. ABA catabolism is regulated through two different pathways: oxidation and conjugation. ABA is usually oxidized in carbon 8′ [19, 20] in a reaction catalyzed by an ABA-specific 8′-hydroxylase that is encoded by a gene of the cytochrome P450 707A gene family [21]. Three genes encoding ABA-specific 8′-hydroxylases (*VvHyd1*, *VvHyd2* and *VvHyd3*) have been characterized in grapevine [22]. Unlike oxidation, ABA conjugation with glucose to form glucosyl ester (ABA-GE) is a reversible process that is catalyzed by an ABA-specific UDP-glucosyltransferase enzyme (UGT) [23, 24]. On the other hand, the hydrolysis of the ABA-GE conjugate is carried out by a β-glucosidase enzyme (BG) [25]. There are three coding genes for BG (*VvBG1*, *VvBG2* and *VvBG3*) in grapevine [26], whereas no reports exist on the number of coding genes for UGT in this species.

In this work, we studied the effect of the sucrose concentration and a semipermeable membrane placed over the differentiation medium on the maturation of stamen filament-derived somatic embryos of grapevine cv. Mencía. To study the regulation of the maturation process, grapevine embryogenic aggregates were cultured under different water stress conditions. We also analyzed the relative expression profile of the *VvNCED1*, *VvHyd2, VvUGT* and *VvBG2* genes using quantitative PCR (qPCR), with a previous determination of the more stable reference genes for normalization, and correlated them with the endogenous levels of ABA and its glucosyl ester (ABA-GE). The effect of exogenous ABA on *VvNCED1* gene expression was also tested as a contribution to the study of the regulation of ABA biosynthesis during the maturation of grapevine somatic embryos.

## Results and discussion

### Effect of sucrose levels and a semipermeable membrane on the grapevine cv. Mencía somatic embryo maturation

**Fig. 1:**
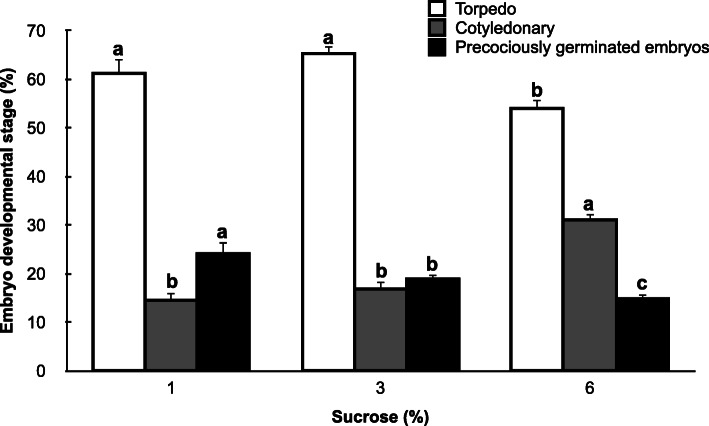
Effect of the sucrose concentration in the DM1 differentiation medium on the developmental stage of grapevine cv. Mencía somatic embryos. Data are shown as percentages (mean ± standard error) of the total number of embryos. Different letters represent significant differences among concentrations of sucrose in the medium for the same developmental stage (Mann-Whitney *U* test, *p* < 0.05)

**Fig. 2:**
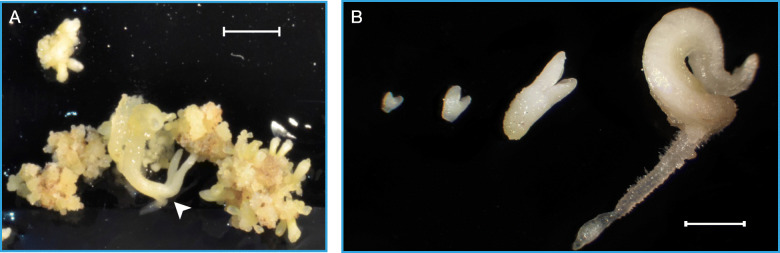
Grapevine cv. Mencía somatic embryo aggregates cultured on DM1 differentiation medium. **a** Somatic embryo aggregates showing asynchronic development after 30 days of culture. Arrowhead: Precociously germinated somatic embryo. Bar: 3 mm. **b** Developmental stages of somatic embryos cultured on DM1 differentiation medium. From left to right, heart stage, torpedo stage, cotyledonary stage, and precociously germinated embryo. Bar: 1 mm

In the present work, we evaluated the effect of sucrose concentration on the development and precocious germination of grapevine cv. Mencía somatic embryos. In grapevine, the media used for somatic embryo differentiation contains at least 3% sucrose [27], and up to 6% in most reports [28–30]. Although we used previously less than 1% with this goal [8], we found here that an increase in sucrose concentration in the differentiation medium led to a significantly higher percentage of mature embryos reaching the cotyledonary stage and a significant decrease in the percentage of precociously germinated embryos (Fig. 1). Similar results have been found in soybean and *Brassica napus* seeds, where the increase in sucrose concentration strongly affected or delayed precocious germination [31, 32]. Although embryo maturation occurred asynchronously (Fig. 2), these results suggest that the maturation of grapevine somatic embryos may be improved by adjusting the osmotic potential of the culture medium.

**Fig. 3:**
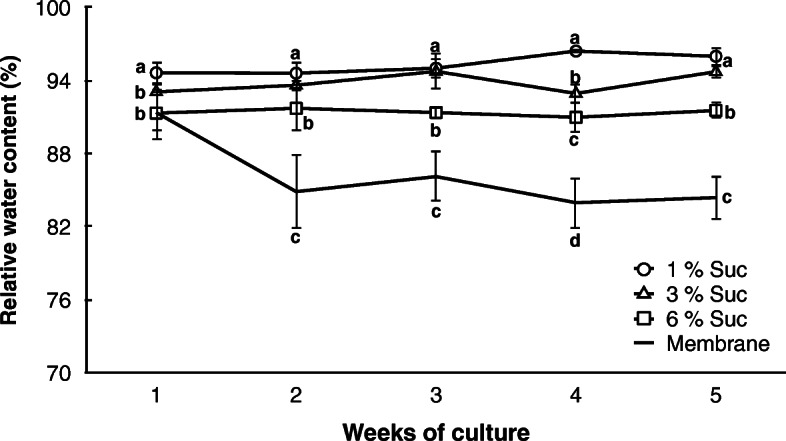
Relative water content of grapevine somatic embryo aggregates cultured for 5 weeks on DM1 solid medium supplemented with different amounts of sucrose (1, 3 or 6%) and over a semipermeable membrane on DM1 medium with 6% sucrose. Values (means ± standard errors) are shown as percentages of water loss ((fresh weight-dry weight) x (100/fresh weight)). Different letters indicate significant differences (Kruskal-Wallis test with Bonferroni post hoc test, *p* < 0.05) among treatments in the same week of culture

**Fig. 4:**
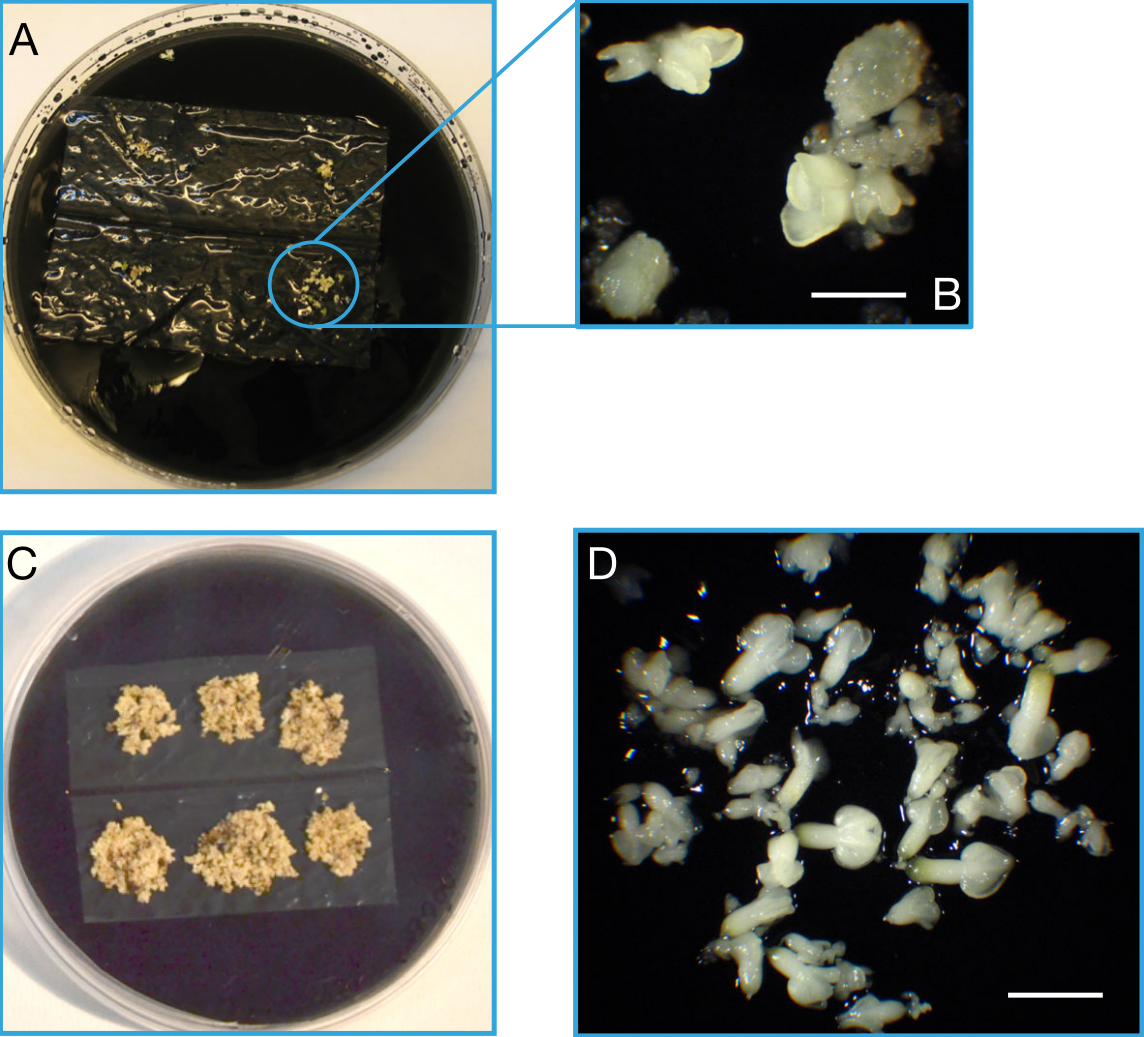
Differentiation of grapevine cv. Mencía somatic embryo aggregates cultured in DM1 medium over a semipermeable cellulose acetate membrane. **a** General view of a 9 cm-diameter Petri plate containing DM1 differentiation medium with somatic embryo aggregates just placed over the semipermeable membrane. **b** Detail of the somatic embryo aggregates cultured on DM1 differentiation medium over the semipermeable membrane. Bar: 0.5 mm. **c** Growth of the somatic embryo aggregates after 30 days of culture on DM1 differentiation medium over the semipermeable membrane. **d** Normal development of somatic embryos on DM1 differentiation medium over the semipermeable membrane. Bar: 1 mm

An increase in the sucrose concentration in the culture medium led to a decrease in the water content of the somatic embryos. Cultures on DM1 medium containing 1% sucrose had the highest water content (approx. 95%), which was stable throughout the 5 weeks of culture (Fig. 3). The water content of the somatic embryo aggregates cultured on 6% sucrose also remained stable but at lower values (approx. 92%) than those in 1% sucrose. The cultures in 3% sucrose showed an intermediate water content between those of the 1 and 6% sucrose cultures. Finally, the introduction of a semipermeable membrane between the somatic embryo aggregates and the culture medium led to a significantly higher loss of water from the embryos starting in the second week of culture (from 91.4 to 84.9%; Fig. 3). Under these conditions (Fig. 4a, b, c), the somatic embryos appeared to develop normally (Fig. 4d) as they did not show any precocious germination (0%), although a detailed quantitative record of the embryo developmental stages was not performed.

Based on these data, we conclude that only the increase of the sucrose concentration at the levels tested is not enough to reach the level of water stress needed to control the maturation of grapevine somatic embryos, which is important for the normal conversion of somatic embryos to plants, as previously shown [8]. In avocado, the use of a semipermeable cellulose membrane during the prematuration step of somatic embryos resulted in mature somatic embryos of better quality and germination ability [14].

### Endogenous ABA and ABA-GE levels in somatic embryo aggregates

**Fig. 5:**
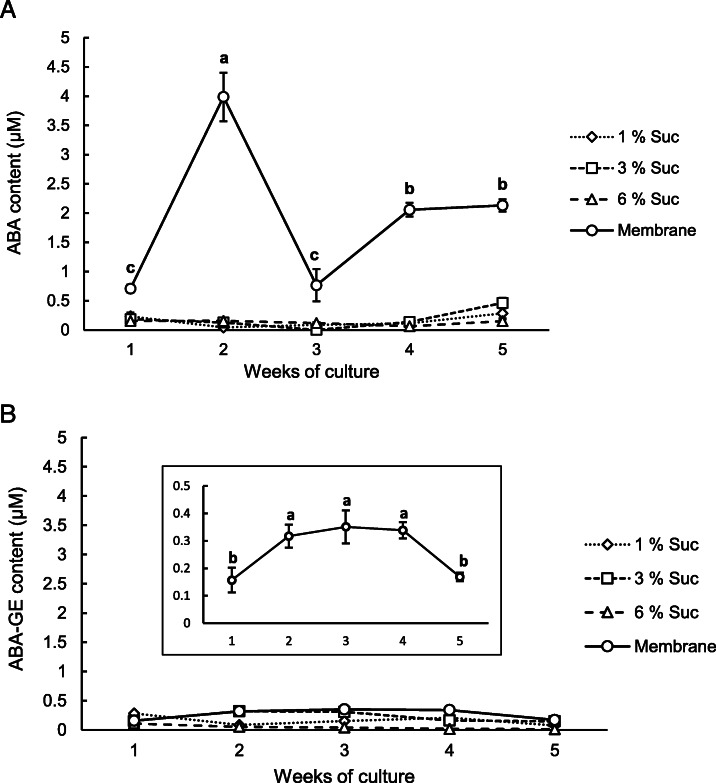
Time course of endogenous concentrations (mean ± standard error) of ABA (**a**) and ABA-GE (**b**) in grapevine cv. Mencía somatic embryo aggregates cultured for 5 weeks on DM1 differentiation medium supplemented with 1, 3 or 6% sucrose and over a semipermeable membrane on DM1 medium with 6% sucrose. The insert in (**b**) shows the time course of the endogenous concentration of ABA-GE in grapevine somatic embryo aggregates cultured for 5 weeks over a semipermeable membrane on DM1 medium with 6% sucrose. Different letters indicate significant differences (Student-Newman-Keuls test, p < 0.05)

In developing embryos, a programmed accumulation of ABA occurs prior to seed desiccation [33, 34]. This suggests that ABA plays a dual role, both suppressing precocious germination of the embryos and stimulating the expression of products associated with the maturation phase [35]. The analysis of the endogenous ABA and ABA-GE levels (Fig. 5) showed that their levels were steadily low (always less than 0.5 μM) in somatic embryo aggregates cultured on DM1 differentiation medium without the membrane and supplemented with increasing levels of sucrose. In these media, no significant changes were observed in the water content of the somatic embryo aggregates (Fig. 3).

In contrast, the somatic embryo aggregates cultured over a semipermeable membrane on DM1 differentiation medium with 6% sucrose showed a nearly eight-fold accumulation of ABA in the second week of culture (Fig. 5a), coinciding with the significant loss of water from the embryos (Fig. 3). After the third week of culture, the endogenous level of ABA in the somatic embryo aggregates was significantly lower than that in the second week, reaching steady values of approximately 4-fold higher than the initial level at the end of the culture period. In addition, significant changes in the endogenous content of ABA-GE in the somatic embryo aggregates cultured over the membrane were observed, with higher values from the second to the fourth weeks (Fig. 5b insert).

In many species, two peaks of ABA accumulation have been documented during seed maturation, both of which contribute to the final level of ABA. The first peak seems to be maternally derived and occurs immediately prior to the maturation phase, whereas the second peak results from internal embryo ABA synthesis [36]. In Norway spruce, a sharp increase in the endogenous levels of ABA and its derivatives in the embryogenic suspensor masses was observed following the passage to maturation medium [4]. The results obtained in this work show a similar ABA content profile (Fig. 5a) in the somatic embryo aggregates differentiated over a semipermeable membrane on DM1 medium; this culture condition prevents precocious germination, which is key for normal, high plant conversion rates [8]. In grapevine, it has been shown that the precocious germination of somatic embryos can be related to their ABA content, which is lower than that in their zygotic counterparts [16]. These results suggest that a certain level of ABA accumulation is necessary for normal somatic embryo development and to prevent precocious germination in grapevine (reviewed in [1, 37]) and that this response is probably linked to the water stress condition induced by the semipermeable membrane.

### Expression of ABA metabolism genes during the differentiation of grapevine somatic embryo aggregates

We tried to better understand the role of water stress and endogenous ABA content in the development of grapevine somatic embryos. No differences in these parameters were observed in somatic embryos cultured in DM1 media supplemented with different amounts of sucrose and without the semipermeable membrane. Hence, the expression levels of genes (*VvNCED1*, *VvHyd2*, *VvUGT* and *VvBG2*) that encode enzymes involved in ABA biosynthesis and catabolism were studied with regard to the effect of the semipermeable membrane.

With this goal, we previously determined the most suitable reference genes for the accurate relative quantification of gene expression levels by qPCR. To perform this, 14 candidate genes (using the 15 primer pairs from Reid et al. [38]) were analyzed using three different statistical procedures (geNorm, BestKeeper and NormFinder). The transcript relative abundance estimated from the quantification cycle (Cq) of the candidate genes ranged from 24 to 30, with most values falling between 24 and 27 (Supplementary Fig. S1). The PCR efficiencies for all candidate reference genes were between 1.92 and 2.00 (92–100%).

All the genes analyzed with the geNorm software [39] presented gene expression stability values (M) lower than 0.9, having *EF1-α(m)* and *GAPDH(m)* the lowest M values (Table 1). As with geNorm, the most stable candidate reference genes ranked by NormFinder [40] were *EF1-α(m)* and *GAPDH(m)* (Table 1). *AP47* (Clathrin adaptor complexes medium subunit family protein) was designated the third most stable using both algorithms.

**Table 1:** Ranking of the candidate reference genes, listed from most to least stable, in the samples from grapevine somatic embryo aggregates cultured in DM1 differentiation medium, as based on the geNorm, NormFinder and BestKeeper software programs

Rank order	geNorm		NormFinder		BestKeeper^a^	
	Gene name	M value	Gene name	Stability value	Gene name	Pearson correlation (r)
1	*EF1-α(m)*	0.346	*EF1-α(m)*	0.115	*UBQ-L40*	0.978
2	*GAPDH(m)*	0.346	*GAPDH(m)*	0.155	*EF1-α(m)*	0.963
3	*AP47*	0.411	*AP47*	0.212	*GAPDH(m)*	0.951
4	*MDH(m)*	0.465	*TIP41*	0.277	*α-Tubulin*	0.934
5	*UBQ-L40*	0.490	*EF1-α*	0.290	*AP47*	0.924
6	*EF1-α*	0.517	*UBQ-L40*	0.316	*UBQ-10(m)*	0.923
7	*TIP41*	0.539	*MDH(m)*	0.341	*MDH(m)*	0.911
8	*α-Tubulin*	0.566	*UBQ-10(m)*	0.374	*EF1-α*	0.905
9	*UBQ-10(m)*	0.588	*SAND*	0.389	*TIP41*	0.847
10	*SAND*	0.620	*α-Tubulin*	0.448	*SAND*	0.785
11	*PP2A*	0.654	*PP2A*	0.478		
12	*Actin*	0.690	*Actin*	0.508		
13	*β-Tubulin*	0.732	*β-Tubulin*	0.656		
14	*UBC*	0.789	*UBC*	0.702		
15	*Cyclophilin*	0.843	*Cyclophilin*	0.725		

^a^BestKeeper is able to analyze a maximum of ten genes; therefore, the five worst-ranked candidates in previous analyses by geNorm and NormFinder (*PP2A*, *Actin*, *β-Tubulin*, *UBC* and *Cyclophilin*) were excluded from the studied data sets

As BestKeeper [41] can only examine up to ten candidate reference genes, the five genes with the lowest rank after analysis with geNorm and NormFinder (Table 1) were not included in this analysis. *EF1-α(m)* and *GAPDH(m)* were among the three most stable candidate genes as determined by BestKeeper, in agreement with the results of geNorm and NormFinder, although *UBQ-L40* (Ubiquitin extension protein 1 (UBQ1)/60S ribosomal protein L40) was ranked first by BestKeeper (Table 1).

The minimum number of reference genes to be used for the normalization of gene expression was determined using the variation by pairs (V) calculated using the software geNorm. The data obtained in this work produced a V value of 0.14, indicating that the use of two genes would be sufficient for accurate normalization [39]. Hence, *EF1-α(m)* and *GAPDH(m)* were selected as the two most stable reference genes in our experimental system. Other genes which were well ranked in Reid’s study [38] in the grapevine were not as stable in this study, supporting the view that the direct extrapolation of the results from a previous reference gene analysis in an experimental system to a different system is not a suitable procedure, even within the same species.

Using *EF1-α(m)* and *GAPDH(m)* as the reference genes for qPCR normalization, we determined the relative expression of the *VvNCED1* (accession codes AY337613.1 and VIT_19s0093g00550), *VvHyd2* (NM_001281052.1 and VIT_02s0087g00710), *VvUGT* and *VvBG2* (XM_002267559.4 and VIT_07s0005g00390) genes during the maturation of grapevine somatic embryos in DM1 medium supplemented with 6% sucrose with and without a semipermeable membrane. As the sequence of the *UGT* gene was not previously described in grapevine, we aligned sequences of *Arabidopsis thaliana* (L.) Heynh. (NM_113074.2), *Pyrus communis* L. (FJ854494.1), *Hevea brasiliensis* (Willd. ex A. Juss.) Müll. Arg. (JQ037843.1), *Nicotiana tabacum* L. (NM_001325655.1) and *Phaseolus vulgaris* L. (KF569682.1), with the goal of determining the conserved regions among them to obtain a sequence of this gene that would be suitable for designing qPCR primers for expression analyses. As a result of conventional PCR experiments, a consensus sequence of 468 bp (XM_003633312.3) with 94% similarity with a *Vitis vinifera* L. sequence (VIT_12s0055g00200) was obtained, and this sequence was used for designing the qPCR primers (Table 2). In addition, this consensus sequence for the putative *VvUGT* gene showed 79% similarity with sequences of the *UGT* gene from *Panax ginseng* C. A. Mey. (KM491309.1), and 74% similarity with the *UGT* gene from *Maclura pomifera* (Raf.) C. K. Schneid. (DQ985178.1).

**Table 2:** Primer sequences, amplicon lengths and efficiency for qPCR assays

Gene description and purpose	Primer sequence (forward / reverse)	Amplicon length (bp)	qPCR^a^ Efficiency
*VvHyd2* PCR	CAAGTATCTGGGCGAAAACC / CATTCTGGGGAAGAGCAAA	509	–
*VvHyd2* qPCR	CATCATAGGCGTCATCTTCG / GGACTCTTGCTCTTCCGTGA	118	1.93
*VvUGT* PCR	TAAATACGTTTATTGAGCTTGA / ACACCGTACCATATGCTTTC	468	–
*VvUGT* qPCR	TCGCTTCTTGTGGTCCCTTC / CCAATTCTAGCCGTCCGATG	120	1.94
*VvBG2* PCR	TCTGGCACCTCTGCTTATCA / CCCTGTTGTGTTTCCTGATACT	644	–
*VvBG2* qPCR	TTTGCTTTGGGTGGTTATGA / GCCAACAAGATGTGATGTGC	125	1.93
*VvNCED1* qPCR	CCTCTGTCTCACAGCAATGGAACT / ATTACCGGCGATTTGCACTCT	140	1.96

^a^ The qPCR efficiency was calculated using LinRegPCR software [42]

ABA content is believed to depend closely on the expression level of the *NCED* gene. This concept is supported by data from ripening avocado (*Persea americana)* fruits, in which the expression levels of *PaNCED1* and *PaNCED3* genes fluctuate similarly to their endogenous ABA levels [43]. This idea is also supported by the fact that overexpression of *NCED* in tomato seeds increased their ABA level and extended their dormancy [44]. Compared with the role of ABA biosynthesis, less is known about the role of ABA catabolism in the regulation of endogenous ABA levels. The expression analyses of the *VvHyd2*, *VvUGT* and *VvBG2* genes performed in this study provide new information about molecular regulation during the maturation of grapevine somatic embryos.

**Fig. 6:**
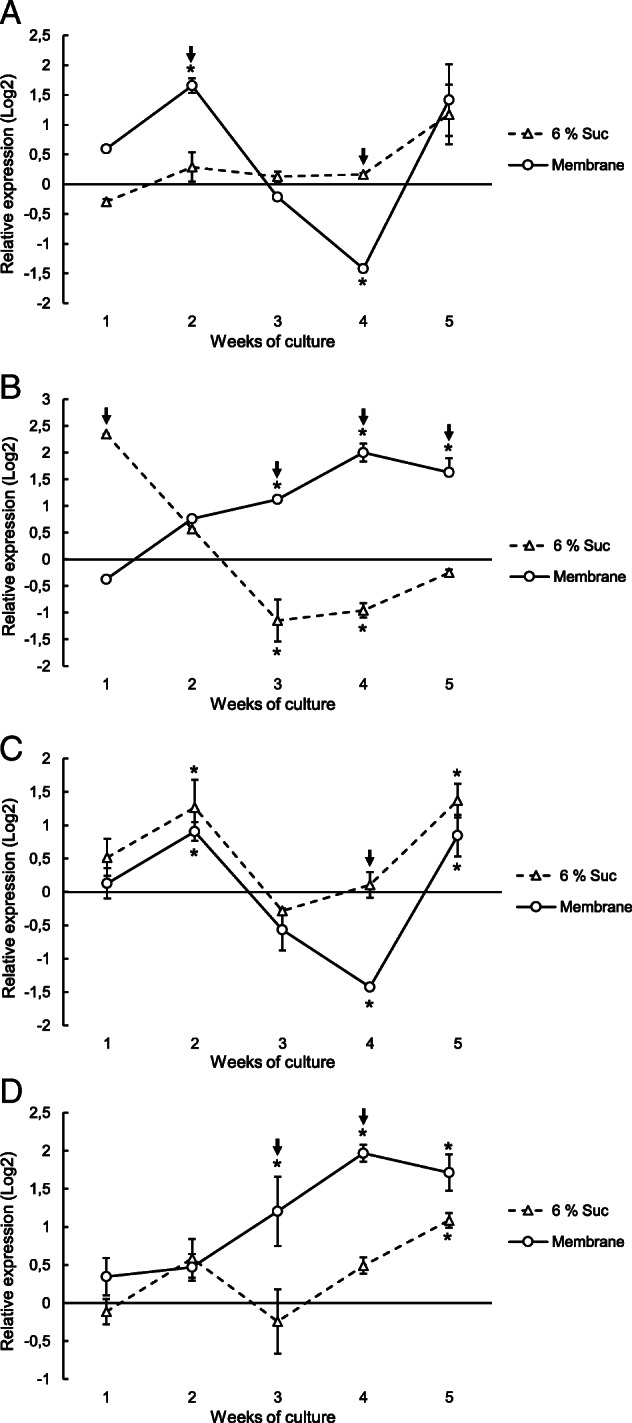
Relative expression profiles of ABA metabolism genes (**a**, *VvNCED1*; **b**, *VvHyd2*; **c**, *VvUGT*; and **d**, *VvBG2*) in grapevine cv. Mencía somatic embryo aggregates cultured for 5 weeks on DM1 differentiation medium supplemented with 6% sucrose or over a semipermeable membrane on the same DM1 medium with 6% sucrose. Somatic embryo aggregates collected just at the time of transfer to DM1 medium (nontreated samples) were used as the calibration group for the relative expression analyses. *GAPDH(m)* and *EF1-α(m)* were used as the reference genes for normalization. Data are represented as the means (± standard error) of two independent experiments. Asterisks indicate statistically significant differences between the calibrator group and the analyzed group, whereas arrows indicate statistically significant differences between treatments with or without the membrane, which in both cases were calculated using REST-2009© software (p < 0.05)

Expression data evaluated by the REST software revealed that the *VvNCED1* gene maintained a low basal level of expression during the culture of somatic embryo aggregates on DM1 medium supplemented with 6% sucrose (Fig. 6a), in which a very low level of ABA was detected (Fig. 5a). In embryogenic aggregates differentiated on DM1 medium without a semipermeable membrane, overexpression of the *VvHyd2* gene was observed during the first week of culture, and was significantly reduced thereafter; it was repressed in the third and fourth weeks and recovered to values near zero in the fifth week (Fig. 6b). This result is in accordance with the low ABA levels observed in the same plant material (Fig. 5). The relationship between endogenous ABA levels and the expression of the *CYP707A* gene family, to which the *VvHyd2* gene belongs, was also observed by Kondo et al. (2012) [45], who determined that the apple *MdCYP707A1* and *MdCYP707A2* genes were repressed after endogenous ABA levels decreased. On the other hand, in grapevine embryogenic aggregates differentiated in DM1 medium, the expression patterns of the *VvUGT* and *VvBG2* genes (Fig. 6c, d), which are involved in the maintenance of ABA levels through ABA-GE synthesis and degradation, were similar. These results are consistent with the low ABA and ABA-GE levels detected in the grapevine embryogenic aggregates that were differentiated in DM1 medium without a semipermeable membrane (Fig. 5).

However, a differential expression profile was observed when the somatic embryo aggregates were cultured over a semipermeable membrane, showing that the *VvNCED1* gene was upregulated in the second week of culture (Fig. 6a), while the expression of the ABA-degrading *VvHyd2* gene was close to zero (Fig. 6b). This differential expression profile corresponded with a period of more intense water loss (Fig. 3) and closely corresponded with the maximum endogenous ABA levels detected (Fig. 5a). This correlation between the transcript levels of the *NCED* gene and the ABA concentration has also been observed in previous works [46, 47]. In addition, the expression of the ABA-deconjugating *VvBG2* gene (Fig. 6d) during the first 2 weeks of culture over the semipermeable membrane of the somatic embryo aggregates was close to zero. On the other hand, a significant increase in the expression of the ABA-conjugating *UGT* gene (Fig. 6c) was detected in the second week of culture, which could help to explain the significant increase in ABA-GE levels by this time of culture (Fig. 5b, insert). Overall, these results support that ABA accumulation by this time of culture could be due to the de novo synthesis of ABA rather than a process of deconjugation from ABA-GE.

The expression of the *VvHyd2* gene increased significantly from the third week of culture of the somatic embryo aggregates over a semipermeable membrane (Fig. 6b), which contributes to explaining the reduction in the ABA content compared to that in the second week (Fig. 5a). This result suggests that, along with the decrease in *NCED1* gene expression (Fig. 6a), an active catabolic process is involved in the maintenance of ABA levels. The role of the *CYP707A* gene (*VvHyd2*), which encodes the 8′-hydroxylase enzyme, in the reduction of ABA levels is well known. In *Arabidopsis*, *CYP707A* gene expression increases significantly after seed imbibition, coinciding with a sharp decrease in the ABA concentration [21]. The increase in *CYP707A1* and *CYP707A3* gene expression has also been related to a decrease in ABA when *Arabidopsis* plants were cultured under high-humidity conditions [48]. Mutant analysis in *Arabidopsis* also supports the regulatory role of the 8′-hydroxylase enzyme on ABA levels. Insertional mutants for the *CYP707A1* [49] or *CYP707A2* [21] genes display 6-fold higher ABA accumulation than the controls. Inhibitors of the enzyme 8′-hydroxylase have been used to increase the levels of ABA in apple [45, 50] and to improve drought tolerance in *Agrostis capillaris* [51].

The conjugation of ABA, which is controlled by the enzyme UGT, is another pathway for reducing ABA levels in plants. *Arabidopsis* insertional mutants for the *UGT71C5* gene presented higher ABA levels than the wild type, while its overexpression lowered the ABA levels and increased those of ABA-GE [52]. In addition, overexpression of the genes *UGT71B6* from *Arabidopsis* [24] and *PvUGT* from bean [53] produced strong ABA-GE accumulation but without affecting endogenous ABA levels. However, in the somatic embryo aggregates cultured over the semipermeable membrane, a repression of *VvUGT* expression was observed after the second week of culture (Fig. 6c). This expression pattern of the *VvUGT* gene suggests that ABA conjugation is not a critical process in the rapid reduction of its endogenous levels during the differentiation of grapevine somatic embryos. Accordingly, the decrease in the ABA content in the third week of culture of the grapevine somatic embryos in DM1 differentiation medium did not seem to be a consequence of substantial ABA conjugation, since a significant increase in ABA-GE was not detected at that time (Fig. 5b).

The expression of the *VvBG2* gene, encoding a *β*-glucosidase (BG) enzyme [25], increased significantly in embryogenic aggregates starting from the third week of culture over the semipermeable membrane (Fig. 6d). The relationship between the overexpression of *BG* genes and the increase in ABA content was observed in grapevine [26, 54, 55] and watermelon [56]. Mutant analysis in *Arabidopsis* showed that the loss of function of these genes results in lower ABA content [25, 57], whereas their overexpression produced higher ABA concentrations in plants that show drought stress tolerance [25, 58]. However, the predicted increase in ABA levels as a consequence of *VvBG2* gene expression after 3 weeks of culture was not observed (Fig. 5a). This could be due to the significantly high expression of the *VvHyd2* gene at the same time (Fig. 6b). Hence, the ABA generated by the BG enzyme would be catabolized by 8′-hydroxylase, a process that would prevent the detection of any ABA accumulation. These results, as well as the repression of *VvNCED1* gene expression detected in the third week of culture (Fig. 6a), could help to explain the decrease in ABA levels despite the increase in *VvBG2* gene expression.

In the fourth week of culture over the semipermeable membrane on DM1 differentiation medium, high expression levels of the *VvHyd2* and *VvBG2* genes (Fig. 6b, d) and low expression of the *VvUGT* gene (Fig. 6c) were observed in the grapevine embryogenic aggregates. These profiles coincided with a second increase in the endogenous ABA content and with a low level of expression of the *VvNCED1* synthesis gene, which was significantly repressed in the fourth week of culture (Fig. 6a). In addition, a significant decrease in the ABA-GE concentration was observed from the third to fifth weeks of culture (Fig. 5b, insert). In *Arabidopsis*, ABA is produced both by de novo biosynthesis and by organelle-specific β-glucosidases in response to abiotic stresses [59], releasing the active compound from glucosyl conjugates. For these reasons, the basal release of ABA from ABA-GE cannot be overlooked. Likewise, the activity of other NCED enzymes potentially contributing to this increase in ABA must also be considered, as it is known that NCED is encoded by the multigenic *NCED* family. This family has at least seven genes in *Arabidopsis* [60], some of which are strongly induced by water stress [61]. In grapevine, three genes (*VvNCED1*, *VvNCED2* and *VvNCED3*) coding for NCED enzymes have been described [62].

Kushiro et al. (2004) [21] observed that *Arabidopsis* plants subjected to dehydration, in which their ABA content increased, showed active expression of the *CYP707A* genes, although at a lower level than that of the *NCED3* synthesis gene. Hence, these authors suggested that ABA accumulation may be the result of subtle differences between biosynthetic and catabolic kinetics. Priest et al. (2006) [24] showed that the 8′-hydroxylation pathway of catabolism is still active in ABA-deficient mutants, suggesting that the *CYP707A* genes display a constant, basal level of expression. Based on these findings, the increase in the endogenous ABA content observed in the fourth week of culture of grapevine embryogenic aggregates (Fig. 5a) could be explained by a holistic analysis of the expression of all ABA metabolism genes. In summary, our results (Fig. 6) showed that, of the genes related to an increase in ABA levels, one (*VvNCED1*, Fig. 6a) was repressed, while another (*VvBG2*, Fig. 6d) was activated. In contrast, of the genes related to a reduction in ABA levels, again, one (*VvUGT*, Fig. 6c) was repressed while another (*VvHyd2*, Fig. 6b) was activated. Thus, subtle differences in the expression of antagonistic genes that control ABA synthesis and degradation could be responsible for the final ABA level.

### Effect of exogenous ABA on the *VvNCED1* gene expression in somatic embryo aggregates cultured over a semipermeable membrane

**Fig. 7:**
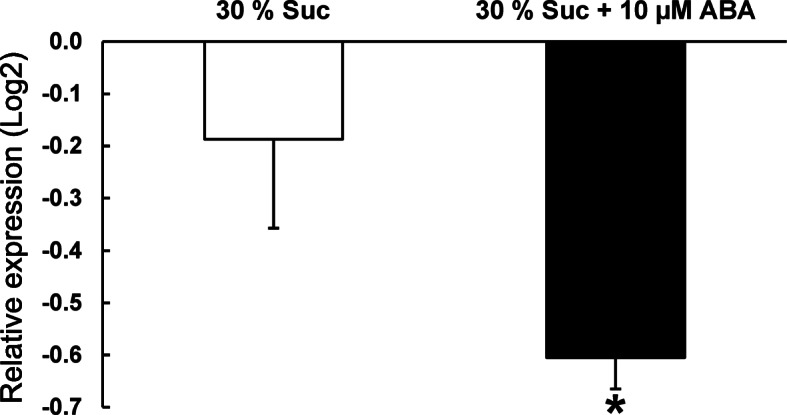
Effect of exogenous ABA on the relative expression of the *VvNCED1* gene in grapevine cv. Mencía somatic embryo aggregates cultured for 2 weeks over a semipermeable membrane on DM1 medium supplemented with 6% sucrose and then treated by pipetting directly onto the aggregates either a solution of 30% sucrose or a solution containing 30% sucrose and 10 μM ABA. The expression levels were calculated relative to the *VvNCED1* gene expression in somatic embryo aggregates cultured under the same conditions but untreated with any solution (calibrator group). *GAPDH (m)* and *EF1-α (m)* were used as the reference genes for normalization. Data are presented as the means of two independent experiments with standard errors. The asterisk indicates a significant difference between the untreated samples and the treated samples (*p* < 0.05), as calculated using REST-2009© software

Finally, we tried to obtain further information on the potential involvement of ABA in the downregulation of the *VvNCED1* gene in the fourth week of culture. To this end, we studied the relative expression level of the *VvNCED1* gene in somatic embryo aggregates cultured over a semipermeable membrane on DM1 medium and treated with exogenous ABA administered in a 30% sucrose solution. Other groups of somatic embryo aggregates were treated with sucrose (30%) as a control to verify that a lack of water stress did not influence the effect of ABA on *VvNCED1* gene expression. The results (Fig. 7) showed a significant downregulation of *VvNCED1* due to the presence of exogenously applied ABA, suggesting that there is a mechanism mediated by ABA to regulate its own biosynthesis during the differentiation of grapevine somatic embryos. Because the *NCED* gene product has been suggested to be the first step performed in the ABA biosynthesis pathway [17], whether this gene is regulated by ABA is a very relevant question. Although there is evidence of an upregulation of *NCED* by ABA [63], it has also been found that *NCED* gene expression was not induced by exogenous ABA in tomato plants [44], and, similarly, that ABA was unable to activate *NCED* genes in cowpea [64]. However, our results presented here suggest that ABA may act as a molecular signal for the regulation of its own biosynthesis.

## Conclusions

Our results support the hypothesis that precocious germination in somatic embryo cultures in grapevine is due to a lack of accumulation of endogenous ABA. Somatic embryo aggregates cultured over a dialysis membrane on DM1 differentiation medium showed a significant loss of water and a complete absence of precocious germination. The analysis of these somatic embryo aggregates revealed a peak of accumulation of endogenous ABA and an upregulation of *VvNCED1* gene expression at the second week of culture, coinciding with significant water loss from the somatic embryos at this time in the culture. In addition, the data from the relative gene expression of ABA-producing (*VvNCED1* and *VvBG2*) and ABA-degrading (*VvUGT* and *VvHyd2*) genes suggested another important point of control of ABA levels during the differentiation of grapevine somatic embryos in the fourth week of culture over the semipermeable membrane. qPCR analysis of *VvNCED1* gene expression in somatic embryo aggregates treated with exogenous ABA showed a significant downregulation of this gene, which suggests the feedback-based control of its expression by the final product of ABA biosynthesis.

## Methods

### Initiation and maintenance of embryogenic cultures and establishment of embryogenic suspension cultures

Inflorescences at stage H on the Baggiolini (1952) [65] phenological scale (corresponding to separated clusters) were collected from adult field-grown plants of *Vitis vinifera* L. cv. Mencía at the Centro de Formación y Experimentación de Viticultura y Enología de Ribadumia (Galicia, northwestern Spain). The late binucleate microspore stage was confirmed with 4′,6-diamidino-2-phenylindole (DAPI, SERVA, Heidelberg, Germany) staining as described by Prado et al. (2010) [8], and inflorescences were washed twice for 5 min with 200 mL of distilled water containing a drop of detergent, chilled at 4 °C for 4 days and then sterilized as described by Kikkert et al. (2005) [66]. Embryogenic cultures were induced from filaments of stamens cultured on induction medium containing Nitsch and Nitsch (1969) [67] salts supplemented with 0.1 μM CoCl_2_, Murashige and Skoog (1962) [68] vitamins, 6% sucrose, 0.1% casein hydrolysate, 1 μM 2,4-D (Duchefa Biochemie, Haarlem, Netherlands) and 4.5 μM thidiazuron (Duchefa). The medium pH was adjusted to 5.8 before autoclaving at 98 kPa and 121 °C, and the medium was solidified using 0.3% Gelrite (Duchefa). Twenty-five stamens per plate were placed on 90-mm-diameter polystyrene Petri plates containing 25 mL of medium. Cultures were maintained under continuous darkness at 24 ± 1 °C and were subcultured onto fresh medium at 30-day intervals.

To initiate embryogenic suspension cultures, 400 mg (fresh weight) of globular somatic embryo aggregates maintained on solid induction medium were transferred into 100-mL Erlenmeyer flasks containing 50 mL of liquid induction medium. The suspensions were incubated on an orbital rotary shaker (150 rpm) at 24 °C in continuous darkness and maintained by replacing 75% of the medium with fresh medium on a weekly basis. After 4 weeks, the suspensions were passed through a 500-μm nylon mesh to obtain a homogeneous suspension composed of embryogenic cell aggregates smaller than 500 μm in diameter, as previously described [7].

### Differentiation of grapevine somatic embryos

The differentiation of the embryogenic cell aggregates into maturing embryos was accomplished by inoculating 200 μL of the embryogenic suspension on 90-mm-diameter polystyrene Petri plates containing 25 mL of DM1 medium consisting of the induction medium described above without phytohormones and supplemented with 0.25% activated charcoal (Duchefa) [8]. Different sucrose concentrations (1, 3, and 6% w/v) were tested in DM1 differentiation medium. The cultures were maintained in continuous darkness at 24 ± 1 °C, and the percentages of embryos at three different developmental stages (torpedo-shape stage, cotyledonary stage, and precociously germinated embryos) were recorded after 4 weeks of culture from four Petri plates per treatment, for about 70 somatic embryos per plate. This experiment was repeated thrice.

To test the effects of the cellulose acetate semipermeable membranes on the differentiation of somatic embryos, globular somatic embryo aggregates maintained on solid induction medium were transferred to dialysis tubing cellulose acetate membranes (Sigma, St. Louis, MO, USA) extended over 25 mL of DM1 medium supplemented with 6% sucrose in 90-mm-diameter polystyrene Petri plates. Six embryo aggregates (approx. 1 g fresh weight) were cultured per plate. The membranes were prepared following the manufacturer’s instructions and were autoclaved twice in distilled water at 121 °C for 20 min. Both fresh and dry weights were recorded weekly over a 5-week period to calculate the relative water content of the embryo aggregates cultured in all differentiation media tested.

### Determination of ABA and ABA-GE

Samples of somatic embryo aggregates cultured in DM1 medium at different sucrose concentrations (1, 3, and 6% w/v) and with 6% sucrose over the semipermeable membrane were collected weekly and frozen in liquid nitrogen. Extracts from three independent samples per treatment were prepared as described by Prado et al. (2014) [9] with some modifications. Fifty milligrams (dry weight) of embryogenic aggregates were homogenized twice with 5 and 3 mL of extraction solvent (methanol/water/formic acid, 75:20:5, v/v/v) containing 0.01% (w/v) butylhydroxytoluene for 16 and 3 h, respectively, by repeated inversion at 4 °C in darkness. Deuterium-labeled internal standards (100 ng of d6-ABA and 100 ng d5-ABA-GE) were added to each of the samples and replicates at the beginning of the extraction procedure as described [69]. The homogenates were cleared by centrifugation (10,000 x *g*, 4 °C, 20 min), the supernatants were combined, and the methanol was removed under a N_2_ stream. Then, formic acid (1 M) was added to adjust the volume to 5 mL, and the extract was applied to a mixed-mode column (Oasis MAX, 150 mg/6 cc; Waters, Milford, MA, USA) preconditioned with 5 mL of methanol and 5 mL of 1 M formic acid. After loading the samples, the column was sequentially washed with 5 mL of 10 mM KH_2_PO_4_ (pH 7) and 5 mL of water. The retained ABA and ABA-GE were eluted by applying 5 mL of 1% (v/v) formic acid in methanol. The solvent was vacuum-evaporated in a Savant Speed-Vac centrifugal evaporator (Thermo Fisher Scientific, Madrid, Spain). The dry fractions were reconstituted in 500 μL of water/acetonitrile/acetic acid (90:10:0.05, v/v) and analyzed using a UPLC Acquity system (Waters) and an API 3000 mass spectrometer (PE Sciex, Concord, Ontario, Canada) following the procedure described by Lopez-Carbonell et al. (2009) [69].

### Total RNA extraction and cDNA synthesis

Three independent samples (biological replicates) of at least 60 mg (fresh weight) somatic embryo aggregates cultured in DM1 medium with and without the semipermeable membrane were collected weekly. The samples were frozen with liquid nitrogen prior to total RNA extraction using the Aurum™ Total RNA Mini Kit (Bio-Rad, Hercules, CA, USA) according to the manufacturer’s instructions. The RNA concentration and purity (260/280 nm and 260/230 nm ratios) were determined with a NanoDrop ND-1000 spectrophotometer (Thermo Fisher Scientific Inc., Waltham, MA, USA) and analyzed on an Agilent 2100 Bioanalyzer RNA 6000 Nano LabChip (Agilent, Mississauga, ON, Canada) to assess the RNA quality. cDNA was synthesized from the total RNA at a ratio of 1 μg per 20 μL reaction volume using the iScript™ cDNA Synthesis Kit (Bio-Rad) according to the manufacturer’s instructions, and reactions were performed on an iQ™ thermal cycler (Bio-Rad).

### Determination of reference genes for qPCR

A set of 15 primer pairs previously used by Reid et al. (2006) [38] in grapevine to target 14 commonly used reference genes was assessed to determine the most stable expressed genes in grapevine embryogenic aggregates cultured on DM1 medium supplemented with 6% sucrose with or without the semipermeable membrane for 5 weeks. Most of these primers amplify a single gene region, except *MDH(m)* (malate dehydrogenase, multitarget), GA*PDH(m)*, UBQ10(m) (polyubiquitin, multitarget) and *EF1-α(m)*, which amplify different regions of members of their gene families. The raw fluorescence data were analyzed using LinRegPCR v.11.0 [42] (available at http://LinRegPCR.nl) to calculate the mean PCR efficiency per primer pair and the Cq value per reaction.

The stability of the reference gene candidates was analyzed using three different software programs: geNorm v.3.4 [39], NormFinder [40] and BestKeeper [41], according to the conditions and restrictions described by the authors. For the geNorm and NormFinder procedures, the Cq values were previously converted to relative quantification data using the ΔCt method. They were performed both with and without the *EF1-α(m)* data as suggested by Reid et al. (2006) [38] to evaluate whether coregulation with *EF1-α* biased the results, considering that *EF1-α(m)* targets *EF1-α* as well as other paralogs. Finally, the two most stable reference gene candidates were selected based on the ranking of stability values validated by the three procedures.

### Primer design and real-time PCR

qPCR primers for the analysis of *VvNCED1*, *VvHyd2* and *VvBG2* relative gene expression were designed from the sequences of grapevine for these genes in the National Center of Biotechnology Information database, and their presence in the genome of the grapevine cv. Mencía used in this study was verified by conventional PCR. As the search retrieved no information regarding sequences for the *UGT* gene in grapevine, the primers for this gene were designed from the alignment of conserved regions of the same gene in other species. Its presence in the grapevine genome was verified by conventional PCR, from which a fragment of 468 bp was sequenced and used for designing the qPCR primers. All conventional PCR and qPCR primers (Table 2) were designed using Gene Runner software (v3.01, Hasting Software Inc., Las Vegas, USA).

The relative abundance of the studied gene transcripts was determined on a weekly basis over 5 weeks of culture of somatic embryos in DM1 medium plus 6% sucrose and with or without a semipermeable membrane. The most stable pair of genes, determined as described above with the geNorm, NormFinder and BestKeeper procedures, were used as the reference genes. Embryogenic aggregates collected at the beginning of culture in DM1 medium were considered the calibrator group. Three biological samples were used per treatment, and each sample was tested in duplicate.

Gene expression analyses were performed following the Minimum Information for publication of Quantitative real-time PCR Experiments (MIQE) guidelines [70]. The qPCR reactions (20 μL), comprising 1X SsoFast™ EvaGreen® Supermix (Bio-Rad), 0.4 μM of each primer and 1.66 ng cDNA were carried out in 96-well plates in an iCycler iQ™ real-time thermal cycler (Bio-Rad). Reactions were performed as follows: 1 min at 98 °C, 40 cycles of 5 s at 98 °C, and 20 s at 58 °C for annealing and extension. Dissociation curves to verify the specificity of each amplification reaction were obtained by heating the amplicons from 65 °C to 90 °C with a ramp setting at 0.5 °C/10 s. Duplicate nontemplate controls were included for each plate.

### Effect of exogenous ABA on *VvNCED1* gene expression during grapevine somatic embryo differentiation over a semipermeable membrane

Globular somatic embryo aggregates (100 mg fresh weight) were cultured for 2 weeks on a cellulose acetate semipermeable membrane extended over 25 mL of DM1 solid medium with 6% sucrose. The cultures were then treated by pipetting a solution of 30% sucrose with or without 10 μM ABA directly to the somatic embryo aggregates 2 days before collecting samples for RNA extraction. Three replicates were performed per treatment, and the experiment was repeated twice. The relative expression levels of *VvNCED1* were analyzed in comparison with the expression level in the somatic embryo aggregates collected at the beginning of culture in DM1 medium over the semipermeable membrane, which were considered the calibrator group. RNA extraction, cDNA synthesis and qPCR were also carried out as described above.

### Data and statistical analyses

All experiments were repeated at least twice independently to ensure the reproducibility of the results. Data on the percentages of grapevine somatic embryos in the different developmental stages were statistically analyzed using a Mann-Whitney *U* test. Data about the relative water content were statistically analyzed using a Kruskal-Wallis test followed by a Bonferroni post hoc test. Data on ABA and ABA-GE contents in the somatic embryo aggregates were statistically analyzed using one-way ANOVA with the Student-Newman-Keuls post hoc test. Statistical tests (*P* < 0.05) were performed using PASW Statistics 18 software (IBM, New Orchard Road, New York, USA).

Data from the qPCR were analyzed using iCycler iQ™ software (Real-Time Detection System Software (Bio-Rad, Windows ver. 3.0). The raw fluorescence data were analyzed using LinRegPCR software [42] to obtain the mean PCR efficiency for each primer pair. Relative gene expression was determined and statistically analyzed (P < 0.05) using the REST-2009© (Relative Expression Software Tool, ver. 2009, [71]) with PCR efficiency correction and normalization by two reference genes as validated for this plant material in the present work, and compared with the 2^-ΔΔCq^ method.

## Supplementary information

**Additional file 1: Supplementary Figure 1.** Box-whisker plot showing Cq variation for the candidate reference genes analyzed in grapevine cv. Mencía somatic embryo aggregates collected at the time of transfer to DM1 medium. The boxes indicate the 25th and 75th percentiles. The whisker caps represent the 10th/90th percentiles. The median is represented by the line within the box, and the outliers are indicated by dots.

## Data Availability

The data and materials used in this study are available from the corresponding author upon reasonable request.

## Abbreviations

2,4-D: 2,4-dichlorophenoxyacetic acid
ABA: Abscisic acid
ABA-GE: ABA-glucosyl ester
*AP47*: Clathrin adaptor complexes medium subunit family protein gene
BG: β-glucosidase
Cq: Quantification cycle
DAPI: 4′,6- diamidino-2-phenylindole
*EF1-α(m)*: Elongation factor 1-alpha gene, multitarget
*GAPDH(m)*: Glyceraldehyde 3-phosphate dehydrogenase gene, multitarget
Hyd: 8′-hydroxylase
IAA: Indole-3-acetic acid
*MDH(m)*: Malate dehydrogenase gene, multitarget
NCED: 9-*cis*-epoxycarotenoid dioxygenase
*PP2A*: Serine/threonine protein phosphatase 2A gene
qPCR: Quantitative PCR
*SAND*: Sand family protein gene
*TIP41*: TIP41-like family protein gene
*UBC*: Ubiquitin-conjugating enzyme gene
*UBQ10*: Polyubiquitin gene, multitarget
*UBQ-L40*: Ubiquitin extension protein 1 (UBQ1)/60S ribosomal protein L40 gene
UGT: UDP-glucosyltransferase
V: Variation by pairs
VP14: Viviparous14
