# Supplementary figures and images for: Changes in abscisic acid metabolism in relation to the maturation of grapevine (Vitis vinifera L., cv. Mencía) somatic embryos
Source: BMC Plant Biol. 2020 Oct 23;20:487. doi: 10.1186/s12870-020-02701-z (PMC7585196; doi:10.1186/s12870-020-02701-z)

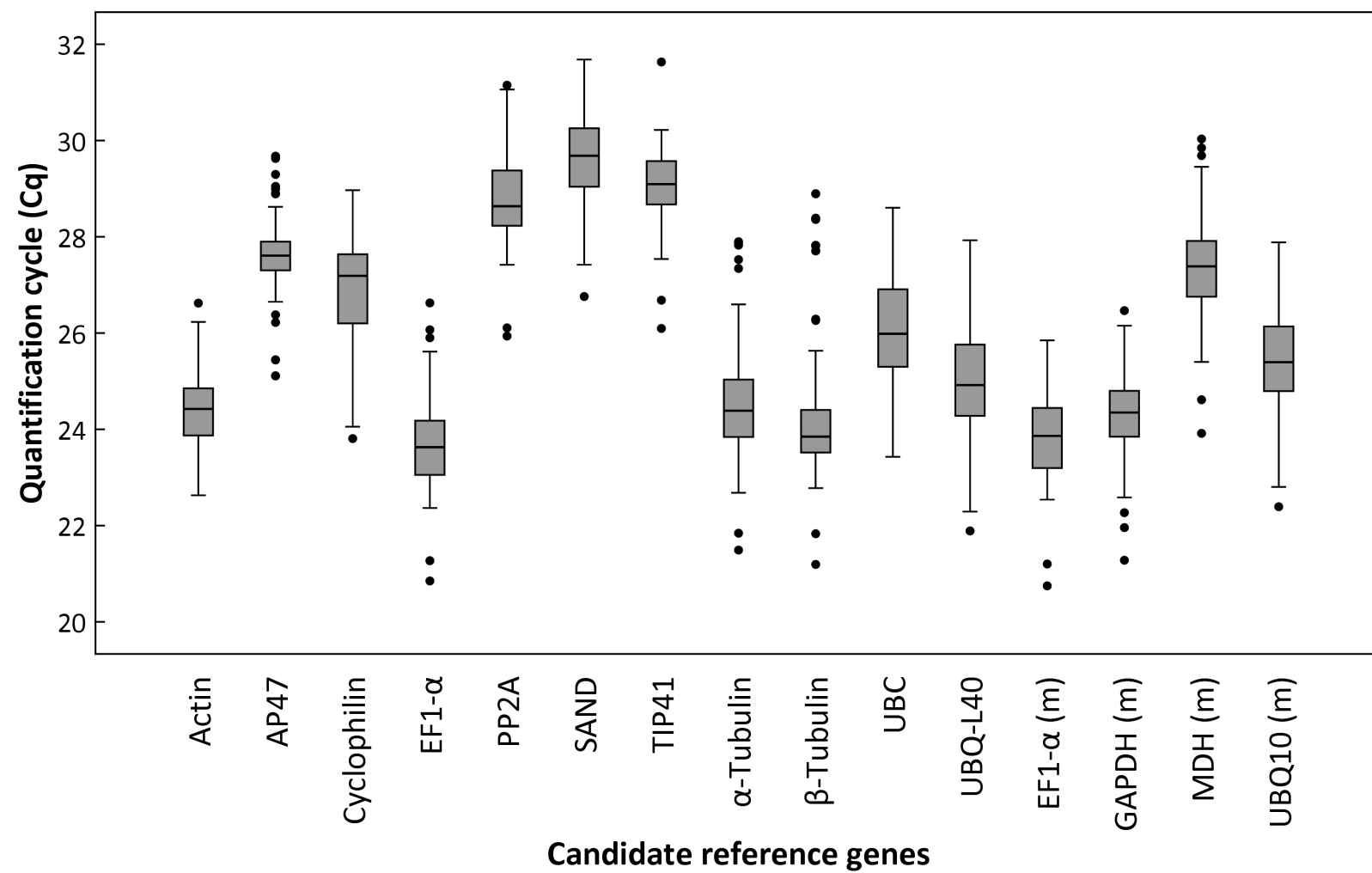

Supplement: Supplementary file 1 — Additional file 1: Supplementary Figure 1. Box-whisker plot showing Cq variation for the candidate reference genes analyzed in grapevine cv. Mencía somatic embryo aggregates collected at the time of transfer to DM1 medium. The boxes indicate the 25th and 75th percentiles. The whisker caps represent the 10th/90th percentiles. The median is represented by the line within the box, and the outliers are indicated by dots. [file 12870_2020_2701_MOESM1_ESM.pdf]
